# Is Blastocyst Culture Responsible for Higher Pregnancy Rates? A Critical Analysis of the Day of Optimal Embryo Transfer and Embryo Quality

**DOI:** 10.5935/1518-0557.20210098

**Published:** 2022

**Authors:** Veronika Günther, Anupama Dasari-Mettler, Liselotte Mettler, Sören von Otte, Johannes Ackermann, Nicolai Maass, Ibrahim Alkatout

**Affiliations:** 1 Clinic for Obstetrics and Gynecology, UKSH Campus Kiel, Arnold-Heller-Straße 3 (Haus C), 24105 Kiel, Germany; 2 University Fertility Center, Ambulanzzentrum des UKSH gGmbH, Arnold-Heller-Straße 3 (Haus C), 24105 Kiel, Germany

**Keywords:** *in vitro* fertilization, embryo quality, day of transfer, blastocyst, pregnancy outcome

## Abstract

**Objective:**

A prolonged culture of embryos beyond day 2-3 to day 5 (blastocyst culture) after fertilization might be an alternative, simple way of selecting suitable embryos for transfer. Extending embryo culture to day 5/6 is a selection tool to choose an embryo with a greater likelihood of implantation rather than improve embryo quality.

**Methods:**

This retrospective study analyzed 1126 fresh IVF/ICSI cycles performed between February 1, 2014 and December 30, 2018 at the University Fertility Center in Kiel, Germany, to determine the impact of blastocyst culture on pregnancy rates and the association between embryo quality and pregnancy rates.

**Results:**

Clinical pregnancy was achieved in 154 cases (19.5%) after day 2/3 transfer and in 76 cases (22.7%) after day 5 transfer. Pearson’s two-sided chi-squared test yielded no statistical significance (p=0.221). The analysis of clinical pregnancy rates in relation to the quality of transferred embryos yielded the following results: 49 (10.7%) pregnancies in cases of no ideal embryo(s); 122 (27.2%) in cases of at least one ideal embryo; and 59 (26.7%) for both quality groups. Pearson’s two-sided Chi-squared test was statistically significant (*p*<0.001).

**Conclusions:**

Our data revealed no improvement of pregnancy rates after blastocyst transfer compared with day 2/3 transfers. However, we noted higher pregnancy rates when an embryo of good quality was transferred.

## INTRODUCTION

Louise Brown, the world’s first in vitro fertilization (IVF) baby, was born on July 25, 1978, in Oldham, England (Steptoe & Edwards, 1978). Initially, IVF was an experimental procedure with very limited outcomes. The first German IVF baby was born at the University Department of Obstetrics and Gynecology in Erlangen in 1982, followed by the University Hospital of Schleswig- Holstein, Lübeck and Kiel, in the same year. Since then, more that 8 million IVF babies have been born all over the world (Kushnir *et al*., 2017). Over the years, there have been numerous developments in stimulation schemes, culture systems, oocyte pick-up methods, embryo transfer, and freezing techniques.

Selecting the “right embryo” is still one of the greatest challenges in IVF (Plachot, 1989). A morphological approach with scoring of the embryos under the microscope in the two- to eight-cell stage, i.e. directly before transfer on day 2-3 after fertilization, has been the method of choice. Earlier studies have shown that morphologically ‘good’ embryos, with few or no anucleate fragments, are prone to having higher implantation rates than others, and seem to be a major predictor of the success of IVF treatment (Ziebe *et al*., 1997; Lundqvist *et al*., 2002). However, the still comparatively low implantation and pregnancy rates of IVF (26.1%) (Nygren & Andersen, 2001) suggest that this approach for early selection on day 2-3 and transfer of embryos needs to be improved by new, more reliable methods.

An alternative and simple way to select embryos for transfer might be to extend the culturing of embryos beyond day 2-3 to day 5 (blastocyst culture) after fertilization. Patients with a high ovarian reserve and several embryos eligible for transfer might particularly benefit from prolonged culture to select embryos. A relatively high proportion of the embryos are developmentally retarded or arrested during prolonged culture, so that pregnancy seems to be achieved faster by choosing the “right” embryo. Successful blastocyst culture depends on successful culture systems. In order to keep an embryo viable in prolonged culturing, appropriate medium is necessary. This medium has to provide for an environment similar to the uterus and thus enable the embryo to develop further. As the embryo cell count increases, the demand for energy carriers becomes more and more differentiated. Due to the loss of pluripotency of the individual cells, energy consumption becomes more complex and the embryo adjusts its energy consumption from pyruvate to the more complex sugar glucose in the first 2-3 days. This finding formed the basis for the introduction of sequential culture, in which culture medium is changed on day 3 (Gardner *et al*., 1998). The most important argument for the change of medium is the adaptation to the physiological conditions of the tubal environment as the site of early embryonic division stages compared to the uterine environment as the site of implantation.

The initial days of embryo development are driven by the maternal genome. The embryonic genome is activated from day 3 onwards. Hence, prolonged culture permits the self-selection of embryos with greater implantation potential as well as accurate assessment. When many cleavage stage embryos are left in culture, only 40% will develop into blastocysts in an average lab, against 60% in an excellent lab (ESHRE Special Interest Group of Embryology & Alpha Scientists in Reproductive Medicine, 2017). In in vivo settings, while cleavage embryos develop in the fallopian tube, the blastocyst is the first stage of the embryo to be exposed to the uterine environment. Hence, the transfer of the embryo into the uterus in the blastocyst stage provides better physiological synchronization between the embryo and the endometrium (Practice Committee of the American Society for Reproductive & Practice Committee of the Society for Assisted Reproductive Technology, 2018), and minimizes the risk of expulsion due to exposure to increased uterine contractility, which is present during the early days (Fanchin, 2001). Blastocysts are known for their better cryo-survival (99%) (Mehta *et al*., 2018).

The purpose of extending embryo culture to day 5/6 is to select an embryo with a greater likelihood of implantation rather than to improve embryo quality. Culture media failed to take into account the changing physiology and requirements of the embryo. It was not until 1996 that a human blastocyst was cultured successfully in vitro. Since then, blastocyst culture has become the standard in the majority of successful laboratories.

The main aim of this study is to investigate the relationship between the day of embryo transfer (day 2/3 vs. day 5) and pregnancy outcome. We also analyzed the relationship between the quality of the embryos transferred (ideal, not ideal, or both) and pregnancy outcomes.

## MATERIAL AND METHODS

A total of 1126 IVF/ICSI (intracytoplasmatic sperm injection) cycles performed between 1 February 2014 and 30 December 2018 at the University Fertility Center in the UKSH (Universitätsklinikum Schleswig-Holstein), Campus Kiel, Germany, were analyzed retrospectively in order to study:

- The impact of blastocyst culture on pregnancy rates; and
- The association between the number of ideal embryos transferred and pregnancy rates.

Patients at the University Fertility Center who had undergone stimulation for oocyte retrieval, oocyte retrieval by transvaginal follicular puncture, fertilization after IVF/ICSI, and an embryo transfer in the same cycle, were eligible for the investigation. Performing an embryo transfer in the same cycle as the stimulation is known as a fresh embryo transfer.

The following patients were excluded from the study:

1. Patients without mature oocytes retrived after stimulation;
2. Patients who did not have a fresh embryo transfer;
3. Patients who had embryo arrest and did not undergo transfer;
4. Patients scheduled for social freezing and fertility preservation;
5. Patients who had ICSI performed with testicular aspirated sperm and not with ejaculated sperm.

Of 1126 ART (assisted reproductive technology) cycles, 791 had a day 2/3 embryo transfer and 335 a day 5 embryo transfer.

We analyzed pregnancy outcomes in relation to the number of ideal embryos transferred. A distinction was made between three categories:

1. No ideal embryos transferred. No embryos of good quality were transferred, although the number of transferred embryos was not taken into account here. In other words, one, two, or three embryos of poor quality may have been transferred.
2. One or more ideal embryos were transferred. Only embryos of good quality were transferred, although the number of transferred embryos was not taken into account here. In other words, one, two, or three embryos of good quality may have been transferred.
3. Both: At least one ideal and one non-ideal embryo were transferred.

**Figure 1:**
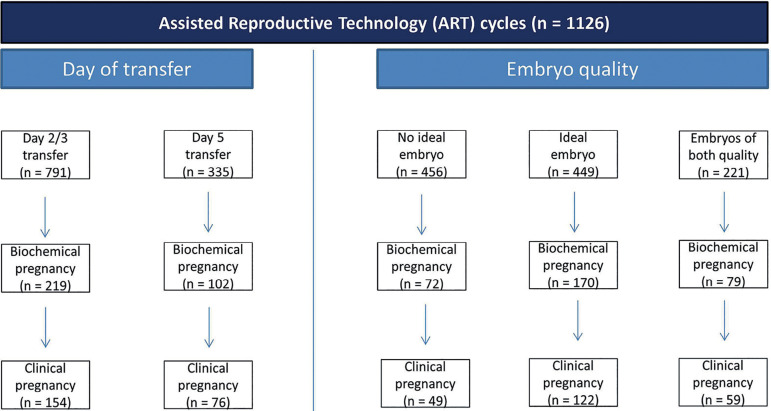
Flowchart: Subdivisions and outcomes of ART cycles.

Figure 1 (day of transfer and embryo quality) and their outcomes in a flowchart.

### Ovarian stimulation and oocyte retrieval

When a couple was selected for an IVF/ICSI cycle, controlled ovarian hyperstimulation for polyfollicular maturation was performed using recombinant follicle-stimulating hormone (FSH) (such as *Gonal-F^©^, Bemfola^©^, Rekovelle^©^, Puregon^©^* or *Ovaleap^©^*) or urinary-derived human menopausal gonadotropin (HMG) (such as Menogon *HP^©^* or *Pergoveris^©^*). The FSH dose (125-450 IU/day) was adjusted according to the patient`s weight, anti-Müllerian hormone (AMH) level, and antral follicle count (AFC). Pituitary down-regulation was achieved with a GnRH antagonist (Orgalutran^©^, 0.25 mg/0.5 ml) from day 6 of stimulation. During follicular monitoring, when at least three of the follicles were ≥ 17 mm in diameter, an hCG trigger of Ovitrelle^©^ (6500 IU) or alternatively Brevactid^©^ (5000/10.000 IU) was given. Transvaginal ultrasound-guided oocyte retrieval either with or without anesthesia was performed 36 hours after triggering with hCG. The embryo transfers were planned for day 2/3 or day 5, depending on previous discussion with the couple and embryo development.

### Laboratory techniques

The cumulus oocyte complexes (COCs) retrieved during follicular aspiration were collected in culture media (GM501 Cult with Gentamicin; Gynemed) in a culture dish (OOPW-CW05-1, Oosafe), and placed in the incubator under strict temperature control, pH, and clean room conditions. The partner’s fresh ejaculated semen sample was processed simultaneously in accordance with a standardized protocol for density gradient (GM501 Gradient 45% and 90%; Gynemed). IVF or ICSI was performed, depending on sperm characteristics. Conventional IVF (25,000 sperms placed on each COC) was performed when the sperm concentration was ≥ 15million and the progressive motility of the sperms was ≥ 32%. In cases with abnormal semen parameters, the COCs were denuded with hyaluronidase (GM501 Hyaluronidase; Gynemed) and graded as immature or mature oocytes. ICSI was performed on mature oocytes using a micromanipulator, while immature oocytes were discarded.

### Embryo quality

Embryos were graded according to the ESHRE consensus workshop (Alpha Scientists in Reproductive Medicine & ESHRE Special Interest Group of Embryology, 2011). Cleavage embryos were defined as good quality (Grade 2) if they had four cells on day 2 and/or 7 or eight cells on day 3, contained <10% anucleate fragments, and exhibited no apparent morphological abnormalities. Poor-quality (Grade 4) embryos included fair-quality (Grade 3) embryos with only 2 cells on day 2, 3-5 cells on day 3 and/or 10-25% fragmentation, and poor-quality (Grade 4) embryos with <3 cells by day 3 and >25% fragmentation (Reinblatt *et al*., 2011; Oron *et al*., 2014).

**Figure 2:**
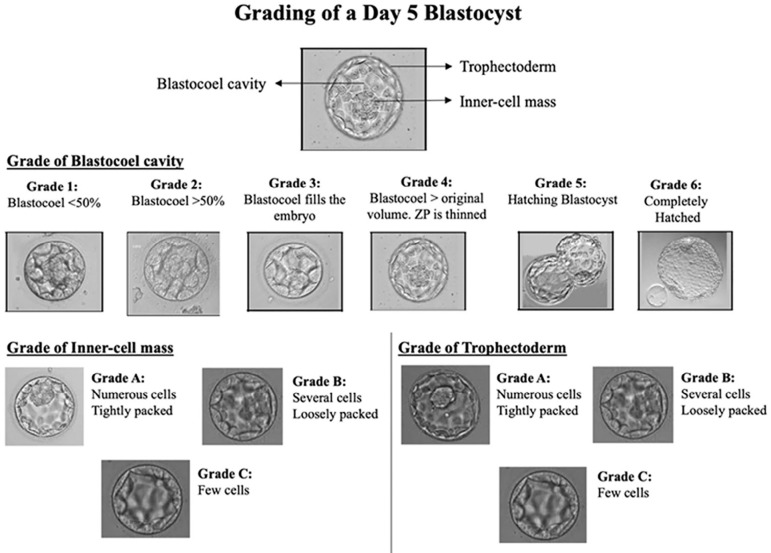
Blastocyst grading scheme of a Day 5 embryo (Blastocyst). ZP: Zona pellucida.

On day 5, the blastocysts were graded as follows. The development of the trophectoderm reflects the embryo’s ability to implant, while the development of the inner cell mass reflects the ability of the fetus to develop (Kovacic *et al*., 2004). The cavity of the blastocyst grows larger during development only if the trophectoderm cells function optimally; this is indirect evidence of embryo competence. Based on the morphological grading of blastocysts (Figure 2), the latter were divided into ideal embryos and non-ideal embryos. Ideal embryos had a blastocoel cavity of minimum grade 3, an inner cell mass of grade B, and a trophectoderm of grade B. Any day 5 embryos not fulyfilling these minimum criteria were regarded as non-ideal. Embryos arrested in development that did not reach the blastocyst stage were discarded.

### Embryo transfer and pregnancy test

On the day of embryo transfer, the cleavage embryos and blastocysts were graded according to the ESHRE consensus workshop (Alpha Scientists in Reproductive Medicine and ESHRE Special Interest Group of Embryology, 2011) and divided into ideal or non-ideal embryos. Embryo transfer was performed under guided ultrasound investigation of the abdomen by an afterload technique: after the clinician had inserted the outer catheter through the patient’s cervix to the inner cervical os, the inner catheter was loaded with the embryos to be transferred and delivered to the clinician by the embryologist. The clinician placed the embryos under abdominal ultrasound guidance at a distance of about 1.5 cm from the fundus of the uterus. Conventional luteal support followed.

Serum levels of beta-hCG were measured 14 days after the transfer to confirm pregnancy. In case of pregnancy, weekly beta-hCG measurements were performed in order to check for adequate increase. The presence of fetal heartbeats on ultrasound examination between the 7^th^ and 9^th^ week of pregnancy was interpreted as clinical pregnancy. Pregnancies failing to continue beyond the positive beta-hCG measurement or ending after a fetal heartbeat had been detected were excluded from the evaluation and assigned to the category of abortions. Thus, biochemical pregnancies and abortions were grouped together.

The majority of patients were followed until delivery, at which time the week of gestation and the infant’s weight, sex, and condition were registered.

### Statistical analysis

Ordinal and nominal data were shown in absolute and percentage frequencies. The relationship between pregnancy outcomes and the number of ideal embryos on the one hand, and the day of embryo transfer on the other, were tested for associations using Pearson’s Chi-squared test. The tests were two-sided; the level of significance was set at 5%. Alpha adjustment for multiple testing was not performed, and the results were interpreted accordingly. SPSS Statistics 26 (SPSS Inc. an IBM Company, Chicago, IL) was used for statistical calculation.

## RESULTS

A total of 1126 ART cycles were included in this retrospective analysis. Table 1 summarizes the demographic data of the patients: age, BMI, number of previous ART cycles (ART attempt number), number of cumulus oocyte complexes retrieved (number of COCs), number of mature oocytes (M2), number of normal fertilized oocytes (2PN), and number of embryos transferred.

**Table 1:** Patient descriptive statistics.

	Mean	Median	Standard deviation	Minimum	Maximum
Age (years)	35.7	36	4.6	23	48
ART attempt number	1.8	1	1.2	1	10
Number of COCs	9.5	9	5.7	1	31
Number of M2	6.9	7	3.5	1	25
Number of 2PN	4.8	5	3.4	1	25
Number of Embryos transferred	1.9	2	0.54	1	3

Abbreviations: COC: Cumulus oocyte complex; PN: Pro-nucleus; IVF: *In vitro* fertilization; ICSI: Intracytoplasmic sperm injection.

Of 1126 ART cycles, 791 were performed on day 2 and day 3 (cleavage stage), and 335 on day 5 (blastocyst stage). Biochemical pregnancy was registered in 219 cases (27.7%) after day 2/3 transfer, and in 102 cases (30.4%) after day 5 transfer. Pearson’s two-sided Chi-squared test yielded no statistical significance (*p*=0.348).

Concerning the association between clinical pregnancy rates and day of transfer, clinical pregnancy was recorded in 154 cases (19.5%) after day 2/3 transfer, and in 76 cases (22.7%) after day 5 transfer. Again, Pearson’s two-sided Chi-squared test revealed no statistical significance (*p*=0.221).

Of 1126 ART cycles, 456 were performed without an ideal embryo, 449 with at least one ideal embryo, and 221 with embryos of both qualities. Biochemical pregnancy was achieved in 72 (15.8%) cases without an ideal embryo(s), 170 (37.9%) cases with at least one ideal embryo, and 79 (35.7%) cases with embryos from both quality groups. Pearson’s two-sided Chi-squared test was statistically significant (*p*<0.001).

**Figure 3:**
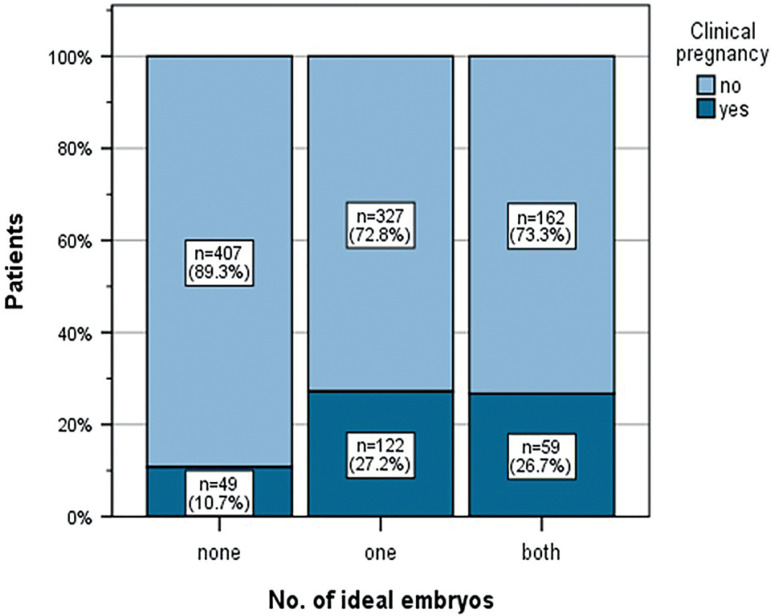
Pregnancy rates depending on embryo quality.

With regard to clinical pregnancy rates in relation to the quality of embryos transferred, the results were as follows: 49 (10.7%) clinical pregnancies in cases without an ideal embryo, 122 (27.2%) in cases with at least one ideal embryo, and 59 (26.7%) in cases of embryos from both quality groups (Figure 3). Pearson’s two-sided Chi-squared test was again statistically significant (*p*<0.001).

Live birth rates were not analyzed in the present report.

## DISCUSSION

The aim of the present study was to investigate pregnancy rates as a function of the day of transfer (cleavage vs. blastocyst stage). We also investigated pregnancy rates as a function of embryo quality (ideal vs. non-ideal). Our data revealed no improvement in pregnancy rates for blastocyst transfers compared with day 2/3 transfers. However, pregnancy rates were increased when embryos of good quality were transferred.

The last decade has witnessed a shift in the ART community towards blastocyst culture, elective single embryo transfer (eSET), and the “freeze all” strategy to improve singleton live birth rates. Initially, ovarian hyperstimulation syndrome (OHSS) was the most feared complication from ovarian stimulation. Following the improved survival of embryos after vitrification, OHSS could be avoided by performing a “freeze all” cycle: after development of the embryos, the best embryos are frozen and transferred in another cycle. Complications of multiple pregnancies could be curtailed through eSET. The success of eSET and the “freeze all” strategy partly depends on successful blastocyst culture. In order to produce 1-2 viable blastocysts, approximately six fertilized oocytes must be left in culture in 85% of the ART cycles (Kliebisch *et al*., 2016). Compared to older women, younger women require fewer fertilized oocytes to be left in culture because the chances of developing a blastocyst mainly depend on age. The decision is a highly personal one.

Finding the correct recombinant follicle-stimulating hormone (rFSH) starting dose in IVF procedures is another important point to discuss and address. Women with polycystic ovarian syndrome (PCOS), particulary the ones with high AMH levels, suffer from the risk of ovarian hyperstimulation syndrome if the rFSH dose is overestimated. Much has been done to identify a correct algorithm, in which the female patient’s age and ovarian reserve markers are considered in the optimization of the rFSH starting dose (Peluso *et al*., 2021).

Di Paola *et al*. (2018) analyzed rFSH doses prescribed in IUI cycles with the help of a nomogram based on female patient age and markers of ovarian reserve. The authors concluded that this nomogram led to a more tailored FSH starting dose in IUI cycles and improved treatment cost-effecitveness. However, these findings appear not to apply to women with PCOS and particularly subjects with high AMH levels (Di Paola *et al*., 2018).

Another study by Burnik Papler *et al*. (2019) evaluated the outcomes of controlled ovarian hyperstimulation in obese women and women of normal weight based on clinical IVF parameters and gene expression in cumulus cells. Obese women required significantly larger amounts of gonadotropins to achieve similar IVF success rates as women of normal weight. Differences in cumulus cells gene expression and smaller proportion of good quality embryos may imply that oocytes derived from obese women are of lower quality. The authors concluded that further studies are needed to evaluate whether obesity itself or the higher doses of gonadotropins used in obese women causes this effect (Burnik Papler *et al*., 2019).

The success of an ART cycle depends on several clinical and laboratory factors, including the couple’s medical characteristics, their clinical reports, and the treatment given (Günther *et al*., 2016). The number of oocytes retrieved, the techniques used in the lab, the development of the patient’s embryos, and the embryo transfer procedure are crucial to the success of ART cycles. Female age is known as a major determinant of the likelihood of pregnancy in an ART cycle (Bhattacharya *et al*., 2013). The presence of uterine fibroids, polyps, adenomyosis, uterine septum, PCOS, dermoids, and other factors also affect outcomes. In male partners, the presence of reduced sperm count (oligozoospermia), sperm motility (asthenozoospermia), sperm morphology (teratozoospermia), or a combination of the three, affect the outcome of ART cycles. The presence of varicocele, hydrocoele, diabetes, and sperm antibodies may also affect outcomes (Fainberg & Kashanian, 2019).

Embryo transfer is a key stage in IVF, in which the quality of performance determines the outcome. According to recent evidence, transvaginal ultrasound guidance of the transfer seems to increase the percentage of pregnancies per transfer, compared with transfers performed under transabdominal ultrasound guidance. Visual monitoring of transcervical passage, which is rendered more precisely and with less trauma, and precision of embryo deposition are factors that probably account for the improvement of pregnancy outcomes (Larue *et al*., 2017). On the other hand, in a systematic review and meta-analysis about this topic, Cozzolina *et al*. found that the evidence supporting the correlations between transvaginal-guided embryo transfer and pregnancy and live birth rates was insufficient, and called for further studies on the subject (Cozzolino *et al*., 2018).

Guerif *et al*. (2009) analyzed pregnancy outcomes after day 2 single embryo transfers (SET) versus blastocyst-stage transfers. The study included 478 couples, of which 243 underwent single embryo transfers on day 2 and 235 single blastocyst transfers (SBT) on day 5/6. The primary outcome was cumulative delivery rate, including fresh and frozen-thawed cycles. The delivery rate per cycle following fresh embryo transfer was significantly higher in the SBT group than in the SET group (*p*<0.01). In contrast, frozen embryo and/or blastocyst transfers tended to result in higher number of deliveries in the SET group compared with the SBT group. Altogether, the cumulative delivery rate per couple, including fresh and frozen embryo transfers, was similar in both groups (37.9% versus 34.2% in the SBT and SET groups, respectively) (Guerif *et al*., 2009).

The present study was confined to patients who underwent fresh embryo transfers. Rather than a single embryo, our patients received one to three embryos. Furthermore, we analyzed clinical pregnancy rates and not delivery rates. Therefore, our results are not entirely comparable with the above-mentioned studies. However, in contrast to the afore-mentioned investigation, we did not achieve higher pregnancy rates after fresh blastocyst transfer.

A Cochrane database comprising 27 randomized controlled trials (4031 couples) compared the efficacy of blastocyst versus cleavage-stage transfers. Clinical pregnancy rates were higher in the blastocyst transfer group after fresh transfer (OR 1.30, 95% CI 1.14 to 1.47; 27 RCTs, 4031 women, I^2^ = 56%; moderate quality evidence). This suggests that if 36% of women achieve clinical pregnancy after fresh cleavage-stage transfer, between 39% and 46% would get pregnant after fresh blastocyst stage transfer.

The Cochrane database has been updated from 2012 onward. Twenty-three randomized controlled trials were included in the older version (Glujovsky *et al*., 2012). No difference in clinical pregnancy rates was noted between early cleavage and blastocyst transfer in the 23 RCTs (Peto OR 1.14, 95% CI 0.99 to 1.32) (day 2 to 3: 38.6%; day 5 to 6: 41.6%). The four RCTs that reported cumulative pregnancy rates (266 women, Peto OR 1.58, 95% CI 1.11 to 2.25) (day 2 to 3: 56.8%; day 5 to 6: 46.3%) significantly favoured early cleavage transfers (Glujovsky *et al*., 2016).

A similar change concerning pregnancy rates over the years has been described by Blake *et al*. (2002; 2005; 2007). Ten randomized controlled trials were included in the Cochrane database of 2002 (Blake *et al*., 2002). No significant difference was noted between the two treatment groups (cleavage versus blastocyst stage) in regard to live birth rates; a difference was only reported in one quasi-randomized trial (Peto OR 1.59, 95% CI 0.80, 3.15). There was also no evidence of a difference in pregnancy rates (overall as well as in subgroups) between the two groups (4 RCTs: Peto OR 0.86, 95% CI 0.57, 1.29). The analysis revealed no overall difference in implantation rates per embryo transferred, although it was impossible to calculate valid confidence intervals from the published data (day 2/3 17.1% vs. day 5/6 18.9%).

The updated Cochrane database of 2005 (Blake *et al*., 2005) included 16 randomized controlled trials. Again, there was no evidence of a difference in live birth rates per couple between the two treatment groups [7 RCTs; OR 1.16, 95% CI 0.74 to 1.44 (day 2/3 34.3% vs. day 5/6 35.4%)] or in clinical pregnancy rates per couple [15 RCTs; OR 1.05, 95% CI 0.88 to 1.26 (day 2/3 38.8% vs. 40.3%)], even for patients with a favorable prognosis (6 RCTs: OR 96% 1.06 CI 0.83 to 1.34).

The updated Cochrane database of 2007 (Blake *et al*., 2007) included 18 randomized controlled trials. A significant difference in live birth rates per couple was noted in favor of blastocyst culture [9 RCTs; OR 1.35, 95% CI 1.05 to 1.74 (day 2/3: 29.4% versus day 5/6: 36.0%)]. This was particularly true of trials consisting of patients with a favorable prognosis, equal numbers of embryos transferred (including single embryo transfer), and trials in which randomization was performed on day 3.

Over the years, the two Cochrane analyses revealed a better outcome after blastocyst transfer. The development of culture media might be one explanation for better results after blastocyst transfer in recent publications.

Published literature contains several reports on culture media and their influence on blastocyst development and pregnancy rates. Jones *et al*. (1998) described a cell-free culture system designed for embryo development to the blastocyst stage. Using this protocol, 52% of zygotes developed to blastocysts and 34 of 35 treated patients received 82 blastocysts and 11 morulae on day 5 or 6. Twenty-one fetal sacs with positive heartbeats (23% implantation rate) were detected in 13 ongoing pregnancies (38% pregnancy rate/ transfer) (Jones *et al*., 1998).

Fawzy *et al*. (2017) analyzed the influence of oxygen concentration in culture media on blastocyst formation rate and clinical outcome. After intracytoplasmic sperm injection (ICSI), the embryos underwent cultivation in either 3.5% O2 concentration (intervention) or 5% O2 level (control group), continuously, from day 0 through day 5 or 6. Significantly higher fertilization and cleavage rates were noted in the 3.5% O2 group [odds ratio (OR) 1.72, 95% confidence interval (CI) 1.53-1.93] than in the 5% O2 group (OR 3.74, 95% CI 2.30-6.07). Culturing embryos in 3.5% O2 concentration led to significantly lower rates of biochemical pregnancy, clinical pregnancy rates, and implantation rates (OR 0.66, 95% CI 0.47-0.92; OR 0.60, 95% CI 0.43-0.84; and OR 0.61, 95% CI 0.46-0.81, respectively).

The authors concluded that culturing human embryos continuously from day 0 to 5 or 6 in 3.5% O2 concentration was associated with significantly higher rates of fertilization and cleavage, but with a significantly poorer outcome in regard to clinical outcome parameters (Fawzy *et al*., 2017).

In order to analyze the impact of culture conditions on blastocyst outcome, Deng *et al*. (2020) compared a single-step medium with a sequential medium and their influence on aneuploidy rates. Interestingly, a higher aneuploidy rate in blastocysts was noted in the single-step medium compared with sequential medium (54.0% vs. 45.8%). There was no difference in clinical pregnancy rates, miscarriage rates, or live birth rates between the two culture media systems when the patients were given a euploid embryo transfer (Deng *et al*., 2020).

The three reports cited above illustrate the changes in culture media over the past decades. A variety of protocols, schemes, and components have led to newer, and supposedly better results in blastocyst cultivation. Changes in study design and individual culture media over the years have made it difficult to compare the results of blastocyst cultures.

The above-mentioned Cochrane analyses have clearly shown the improved outcome of blastocyst transfer over the years, which may have been due to better cultivation media.

We performed no subgroup analysis of the individual years of the study with reference to the different culture media. This might explain the absence of a significant difference between day 2/3 and day 5 transfers. Blastocyst transfer may have only improved in the last few years.

Oron *et al*. (2014) published a study concerning embryo quality and pregnancy outcomes. In 1541 fresh single embryo transfers (SETs), the authors compared pregnancy outcomes resulting from the transfer of a single fresh good-quality (Grade 2) embryo vs. the transfer of a single poor-quality (fair, Grade 3; or poor, Grade 4) embryo. Of all embryo transfers, 1193 involved good-quality embryos and 348 poor-quality embryos. Higher clinical pregnancy (41.5%) and live birth rates (32.3%) were noted in the good-quality embryo transfer group compared with the poor-quality embryo transfer group (19.2 and 15.5%, respectively; *p*<0.0001). There was no significant difference in miscarriage rates between transfers of a single good or poor quality embryo. Multivariate logistic regression analyses for pregnancy complications showed no increased risk of maternal or neonatal complications after the transfer of a poor-quality embryo (Oron *et al*., 2014).

Reports of higher pregnancy rates after transfer of an embryo of good quality are in line with the results of our study. We also registered a significantly higher pregnancy rate after the transfer of at least one ideal embryo. In contrast to Oron *et al*. (2014), we did not perform single embryo transfers alone, but transferred up to three embryos.

Based on a similar study design as ours, Aldemir *et al*. (2020) studied 2298 fresh IVF/intracytoplasmic sperm injection (ICSI) cycles with two good-quality embryos (group A, double embryo transfer, DET), one good, and one poor-quality embryo (group B, double embryo transfer, DET), and single good-quality embryo (group C) transfers. Clinical pregnancy and live birth rates were the primary outcomes. In the cleavage-stage transfer subgroups, clinical pregnancy rates were lower in the single-embryo transfer (SET) subgroup compared with DET subgroups, but the difference was not statistically significant compared to DET with mixed-quality embryos. Live birth rates were similar in the three groups. In the blastocyst transfer subgroups, clinical pregnancy and live birth rates were significantly higher in DET with two good-quality embryos than DET with mixed-quality embryos and SET groups. Multiple pregnancy rates were higher in both DET groups with regard to the day of transfer (*p*=0.001). DET with mixed quality embryos results in lower clinical pregnancy and live birth rates compared with DET with two good-quality embryos in the blastocyst stage. Transfer in the cleavage stage revealed no difference in live birth rates between the two groups.

These data concur with our own: we also noted a higher clinical pregnancy rate after the transfer of good-quality embryo(s) rather than mixed-quality embryos.

## CONCLUSION

The last decade has witnessed a shift in the ART community towards extended embryo culture (day 5, blastocyst culture) and the transfer of human embryos in IVF/ICSI cycles instead of traditional day 2/3 transfer (cleavage stage). The purpose of this shift was to improve pregnancy rates.

We analyzed the pregnancy outcomes of 1126 ART cycles performed from 2014 to 2018 at the University Fertility Center of UKSH, Kiel. Our data revealed no general improvement in pregnancy rates for blastocyst transfer compared with day 2/3 transfer. However, we noted a higher pregnancy rate when an embryo of good quality was transferred. As recent studies have shown, blastocyst transfer has led to higher pregnancy rates. The improvement of culture media might be one explanation for this phenomenon. Future studies with a subgroup analysis of years might yield data concerning a potential increase in pregnancy rates due to scientific progress. A new analysis of pregnancy outcomes might be started when there is any change in culture conditions, such as the culture protocol, culture medium, incubator, or other components. Although this would result in smaller individual groups, it would also eliminate culture conditions as potential factors influencing outcomes.

### Ethics approval and consent to participate

All procedures involving human participants were performed in accordance with the ethical standards of the institutional and/or national research committee, as well as the 1964 Helsinki Declaration and its later amendments or comparable ethical standards. Informed consent was obtained from all participants of the study.

## Abbreviations

IVF: in vitro fertilization
ICSI: intracytoplasmatic sperm injection
ART: assisted reproductive technology
COC: cumulus oocyte complex
eSET: elective single embryo transfer
SBT: single blastocyst transfer
DET: double embryo transfer
